# Structural, Morphological, Optical and Magnetic Studies of Cu-Doped ZnO Nanostructures

**DOI:** 10.3390/ma15228184

**Published:** 2022-11-17

**Authors:** Shalendra Kumar, Faheem Ahmed, Naushad Ahmad, Nagih M. Shaalan, Rajesh Kumar, Adil Alshoaibi, Nishat Arshi, Saurabh Dalela, Fatima Sayeed, Kavita Kumari

**Affiliations:** 1 Department of Physics, College of Science, King Faisal University, P.O. Box 400, Al-Ahsa 31982, Saudi Arabia; 2 Department of Physics, University of Petroleum & Energy Studies, Dehradun 248007, India; 3 Department of Chemistry, College of Science, King Saud University, P.O. Box 2455, Riyadh 11451, Saudi Arabia; 4 Physics Department, Faculty of Science, Assiut University, Assiut 71516, Egypt; 5 University School of Basic and Applied Sciences, Guru Gobind Singh Indraprastha University, New Delhi 110078, India; 6 Department of Basic Sciences, Preparatory Year Deanship, King Faisal University, P.O. Box 400, Al-Ahsa 31982, Saudi Arabia; 7 Department of Pure & Applied Physics, University of Kota, Kota 324005, India; 8 Basic Science Department, Pre-Professional Program-Female, College of Science and Health Profession, King Saud bin Abdul Aziz University for Health Sciences, Al-Ahsa 3660, Saudi Arabia; 9 School of Materials Science and Engineering, Changwon National University, Changwon 51140, Republic of Korea

**Keywords:** ZnO, XRD, DMS, TEM, Optical properties

## Abstract

In the present work, Cu-doped ZnO nanostructures (Cu% = 0, 1, 5) have been prepared using microwave-assisted chemical route synthesis. The synthesized nanostructures were investigated through structural, morphological, optical, and magnetic characterizations. The results of the X-ray diffraction (XRD), high resolution transmission electron microscopy (HR-TEM), and selective area electron diffraction (SAED) patterns confirmed that all of the samples exhibit the single-phase polycrystalline hexagonal crystal structure. The XRD results infer a decrease in the lattice parameters (a/c) by increasing the Cu% doping into ZnO. The field emission scanning electron microscopy (FE-SEM) and energy dispersive x-ray (EDX) spectroscopic measurements revealed the formation of nanostructures, showing the major elemental presence of Zn and O in the samples. The photoluminescence (PL) spectra exhibited photoemission in the UV and blue-green regions. With the increase in the Cu%, the photoemission in the UV region is reduced, while it is enhanced in the blue-green region. Raman spectra of the Cu-doped ZnO nanostructures displayed a blue shift of the $E_{2}^{\mathrm{High}}$ mode and an increase in the peak intensity of E_1_(LO), indicating the doping of Cu ion in the ZnO lattice. The dc magnetization measurements demonstrated the ferromagnetic behavior of all of the samples with an enhanced ferromagnetic character with increasing Cu%.

## 1. Introduction

Over the last few decades, lots of work has been carried out by various research groups on oxide-based magnetic semiconductors because of their enormous potential applications in various fields of science and technology [1,2,3,4,5,6]. Numerous theoretical and experimental groups have studied the physical (such as optical, magnetic, electronic structural, etc.,) and chemical properties (such as the photocatalytic and anti-bacterial ones) of these magnetic semiconductors and reported that the stated properties can be tailored by the minute doping of the transition metals (TM) in the host matrices. Until now, various un-doped and TM-doped oxide semiconductors such as ZnO, TiO_2_, SnO_2_, In_2_O_3_, HfO_2_, and CeO_2,_ etc., have been explored to study the physical and chemical properties, etc., [7,8,9,10,11,12] that are relevant to various applications such as spintronics, spin valve transistors, microwave devices, spin light emitting diodes, non-volatile memory, logic devices, and optical isolators, etc. [4,13,14]. Although, it is noteworthy to mention here that magnetism has been reported in various TM-doped semiconductors, but the results are still changeable, and the mechanism of ferromagnetism (FM) is also debatable.

Among the numerous compound semiconductors, we have chosen ZnO as the host matrix which is most stable one when it is crystallized in a wurtzite structure. ZnO, as an n-type II-VI semiconductor, is a very interesting material due to its unique properties such as it having a direct band gap energy (3.37 eV), a large exciton binding energy (60 meV), an optical transparency, and a chemical stability. Additionally, the doping of numerous impurity elements in ZnO has been widely investigated because it improves its electrical, optical, and mechanical properties, which facilitate its development in many technological applications such as electronic and optoelectronic devices, ultraviolet light-emitting diodes, ultraviolet lasers, and solar cells, etc. [9]. However, it is worth mentioning that these properties of un-doped and doped ZnO significantly depend on the growth mechanism. Various growth techniques such as the sol-gel, co-precipitation, hydrothermal, auto-combustions, microwave hydrothermal ones, etc., have been utilized to develop the ZnO nanostructures [15,16,17]. Herein, we have grown the Cu-doped ZnO nanostructures using the microwave-assisted hydrothermal method and studied their structural, optical, and magnetic properties. It is worth discussing here that there are numerous reports in the literature where many research groups have reported the debatable optical and magnetic properties of un-doped and TM-doped ZnO nanostructures. These reports suggest that the ferromagnetism in TM-doped ZnO may result due to the formation of a secondary phase or cluster. Due to these issues, we have chosen Cu ions as a dopant in the ZnO host matrix because Cu is one of the important transition metals with an [Ar] 3d^10^ 4s^1^ configuration. The unfilled electronic configuration of Cu can introduce unoccupied electronic states in the otherwise fully filled Zn. This can bring interesting modulation to the physical and chemical properties of the ZnO. Further, it is well known that neither metallic Cu nor its oxides (CuO and Cu_2_O) are ferromagnetic in nature. Therefore, the ferromagnetic ordering observed in Cu-doped ZnO will certainly be the intrinsic property of the material. Regardless of the above fact, there are still various controversial results that have been reported in Cu-doped ZnO. Numerous conflicting results are discussed in the literature where some groups have ruled out the presence of FM, whereas others have confirmed FM ordering in the materials [18,19]. Sukumaran et al. synthesized Y-doped ZnO thin films and observed the ferromagnetic ordering at room temperature [20]. Buchholz et al. fabricated Cu-doped ZnO thin films using the pulsed laser deposition method. They reported that n-type ZnO showed the non-magnetic and p-type ZnO ferromagnetic ordering at room temperature with a Curie temperature above 350 K [21], inferring that the p-type carriers are essential to achieve the ferromagnetic order at room temperature. Hou et al. reported on the magnetic properties of n-type Cu-doped ZnO thin films that were prepared using direct current reactive magnetron sputtering. The authors demonstrated that 2% Cu-doped ZnO film showed a magnetic moment of 1.8 μ_B_/Cu with a transition temperature of about 350 K [22]. Furthermore, Agarwal et al. prepared the un-doped and Cu-doped ZnO thin films using the neutral beam sputtering technique. They observed that the un-doped and Cu-doped films exhibit ferromagnetic ordering at room temperature [23]. In this work, we have demonstrated the structural, optical, and magnetic properties of un-doped and Cu-doped ZnO nanostructures. The structure analysis inferred the single-phase synthesis of all of the samples with a ZnO wurtzite structure without any impurity phases. The DC magnetization measurements revealed the ferromagnetic ordering at room temperature.

## 2. Experimental

The microwave irradiation technique was used to prepare the Cu-doped ZnO nanostructures. In this typical synthesis, we used copper acetate (Cu(CH_3_COO)_2_·1H_2_O; 99.999%), Zinc acetate ((Zn(CH_3_COO)_2_·2H_2_O; 99.999%) and potassium hydroxide (KOH; 99.99%). All of the reagents that were used in the synthesis were of analytical grade, and they were purchased from Sigma Aldrich and consumed as received from the company without any additional treatment. A domestic microwave oven (Samsung, Suwon, Republic of Korea, 750 W) was utilized for the preparation of the Cu-doped ZnO nanostructures [24]. In this synthesis, all of the reagents were weighed in desired stoichiometric amounts, and then, they were dissolved in 100 mL of distilled water in a round-bottom flask to make a solution of 0.6 M. The molar ratio of ((Zn(CH_3_COO)_2_·2H_2_O; (Cu(CH_3_COO)_2_·1H_2_O) to KOH was kept at 1:20 for all of the samples. Finally, the solution was kept inside the microwave oven, and the microwave was operated for irradiation at 300 W (irradiation 17 s, stop 12 s) for 20 min. After the irradiation, all of the samples were cooled to room temperature, and thereafter, the precipitates were washed with deionized water and ethanol and separated using a centrifugation technique. In the end, all of the samples were dried in a hot air oven at 80 °C for 24 h. A Phillips X’pert (MPD 3040, Amsterdam, The Netherlands) with a Cu Kα source (λ = 1.54Å) diffractometer was utilized for the crystal structure analysis. The surface morphology of the Cu-doped ZnO nanostructures was captured using a field emission electron microscope (FESEM, JSM-7500, JEOL, Tokyo, Japan).The High-resolution transmission electron microscopy (HR-TEM) images and the selected area electron diffraction (SAED) pattern were recorded using an FE-TEM (JEOL/JEM-2100F version) that was operated at 200 kV. The PL measurements were carried out using a luminescence spectrometer (JASCO, FP-6500, Madrid, Spain) with a Xenon lamp as the excitation source at room temperature. The excitation wavelength that was used in the measurement was 325 nm. The UV-vis spectra were obtained using Model LAMBDA 35, PerkinElmer (Waltham, MA, USA). The Raman spectroscopy measurements were carried out using a Raman spectrometer (NRS-3100) from SINCO Instrument Co. (Seoul, Republic of Korea) at room temperature. The magnetic properties were studied using a commercial Quantum Design Physical properties measurement system (PPMS).

## 3. Results and Discussion

**Figure 1:**
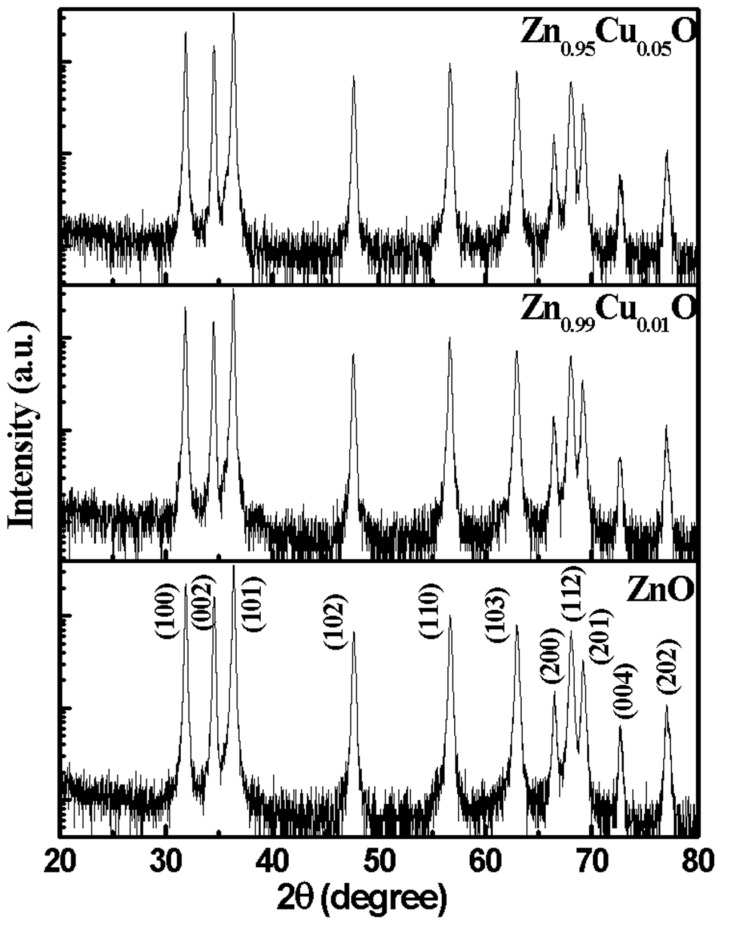
XRD pattern of Cu-doped ZnO nanostructures.

**Figure 2:**
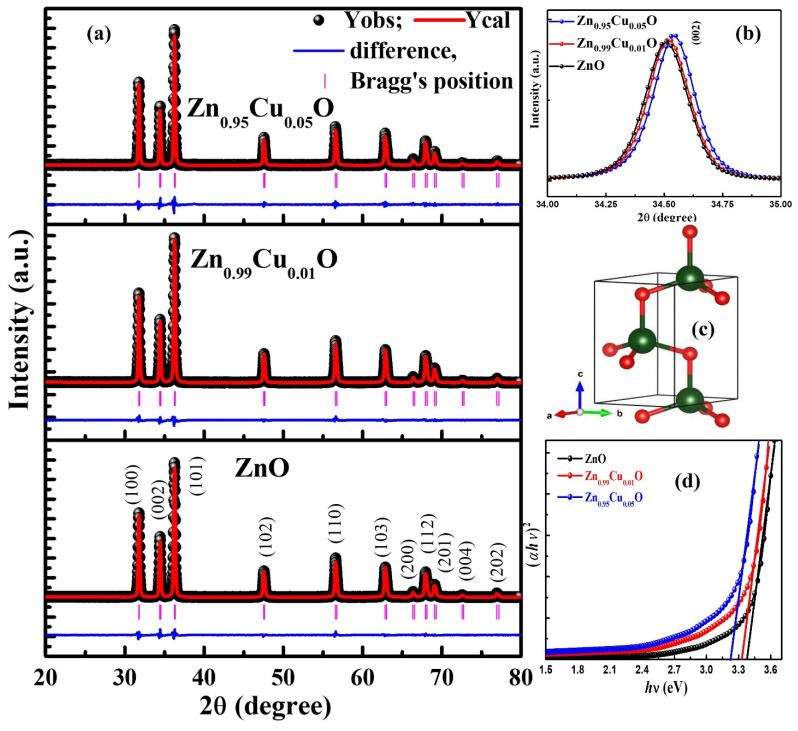
(**a**) Rietveld profiles of XRD patterns of Cu-doped ZnO nanostructures. Observed profiles are shown by the black spheres and calculated profiles are shown by red lines. The pink color vertical sign denotes the Bragg reflections and difference plot is shown blue line. (**b**) Peak shifting (002) plane of Cu-doped ZnO nanostructures. (**c**) Representation of unit cell structure of ZnO nanostructures; green (bigger spheres represent the host atom ‘Zn’, while the small spheres in red color denote the O atoms). (**d**) PL spectra Cu-doped ZnO nanostructures (λ_exc_ = 325 nm).

The phase analysis of the Cu-doped ZnO nanostructures was carried in the θ−2θ mode using the XRD measurements, and these are displayed in Figure 1. To detect the presence of any impurity phases, the XRD data are plotted in the log scale (see Figure 1) which clearly ruled out the presence of any extra diffraction peaks. All of the peaks such as at (100), (002), (101), (102), (110), (103), (200), (112), (201), (004), and (202) which can be observed in diffraction pattern clearly suggests that the Cu-doped ZnO nanostructures are polycrystalline in nature. Additionally, all of the peaks that can be observed in the XRD pattern correspond to the hexagonal wurtzite phase and are in agreement with the JCPDS card number 36–145 [25]. Figure 2a displays the Rietveld refinement XRD pattern where the black points represent the experimentally observed data points and the red color solid line highlights the theoretically calculated pattern. The difference between the experimental and theoretical data is displayed by the blue color. The vertical lines that are shown by the pink color represent the Bragg’s positions. Figure 2b shows the (002) peaks of Cu-doped ZnO nanostructures, which infer that the peaks shifted towards the higher 2θ values with the Cu doping. The shift in the (002) peaks indicates the decrease in the lattice parameters [6]. Therefore, from the XRD data, it can be clearly stated that the Cu ions have successfully replaced the Zn ions in the ZnO host matrix. Furthermore, the crystallographic parameters of the Cu-doped ZnO are determined by the Rietveld refinement method using the FullProof Suite software [26]. Figure 2c represents the unit cell structure of the ZnO nanostructures. The various crystallographic parameters such as the lattice parameters (a, and c), the lattice volume (V), and the discrepancy parameters (χ^2^, R_p_, R_exp_, and R_wp_) which were determined from the Rietveld refinement are tabulated in Table 1. The lattice parameters *a* and *c* have been found to decrease from 3.2506 Å to 3.2500 Å and 5.2070 Å to 5.2058 Å, respectively, with an increase in the Cu doping in the ZnO. The unit cell volume decreased from 47.6499 Å^3^ to 47.6207Å^3^. The decrease in the lattice parameters and lattice volume may have occurred because of the difference in the effective ionic radius of Cu^2+^, and this may be considered as an additional evidence for Cu residing at the Zn sites in the ZnO matrix. The ionic radii of the Cu^2+^ ions (0.73 Å) are somewhat smaller than that of Zn^2+^ ions (0.74 Å). Similar results have also been reported by Ahmed et al., where the Co^2+^/Co^3+^ ions having smaller ionic radii than those of Zn^2+^ have been found to replace the Zn^2+^ ions [5].

**Figure 3:**
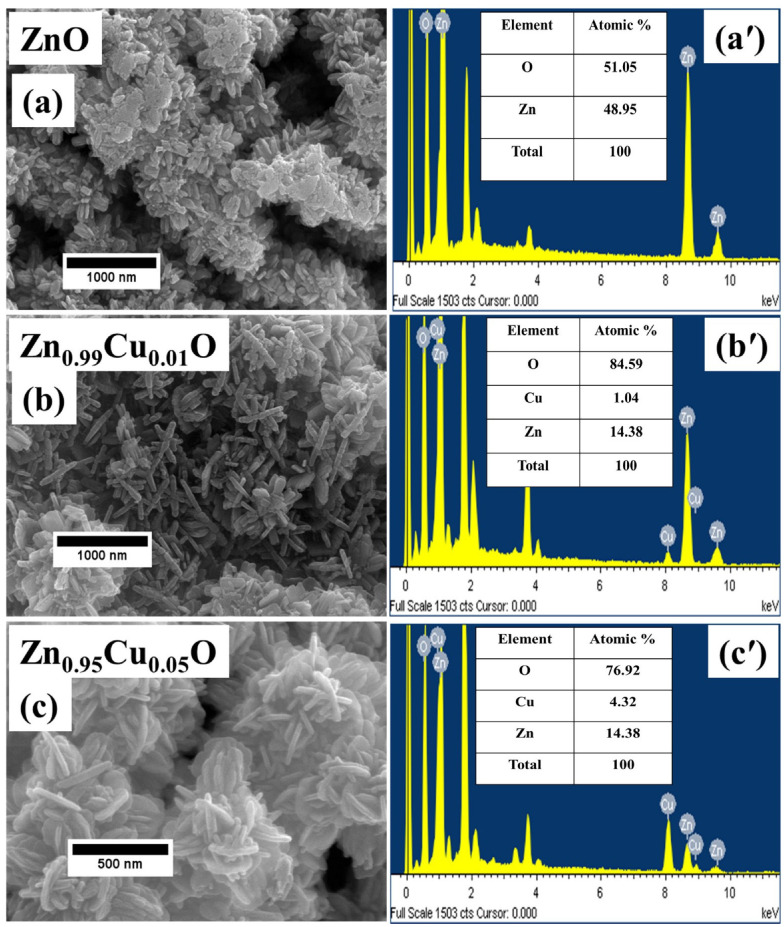
(**a**–**c**) HR-FESEM micrographs of Cu-doped ZnO nanostructures, (**a**′–**c**′) EDX spectra Cu-doped ZnO nanostructures.

Figure 3a–c represents the FE-SEM images of the Cu-doped ZnO nanostructures. It can be seen from the FE-SEM measurements that un-doped and Cu-doped ZnO showed a rod-like morphology. The FE-SEM images demonstrate the formation of a large number of uniform nanostructures with diameters of 50–70 nm and lengths of 300–400 nm. The elemental composition of the Cu-doped ZnO nanostructures which was determined by the energy dispersive X-ray spectroscopy (EDX) are displayed in the inset in Figure 3a′–c′. The EDX measurements infer the presence of Zn and O in un-doped spectrum and Cu, Zn, and O peaks in the 1% and 5% Cu-doped ZnO spectrum. The presence of Cu confirms the growth of Cu-doped ZnO nanostructures and it also confirms the incorporation of Cu in the ZnO host matrix. 

**Figure 4:**
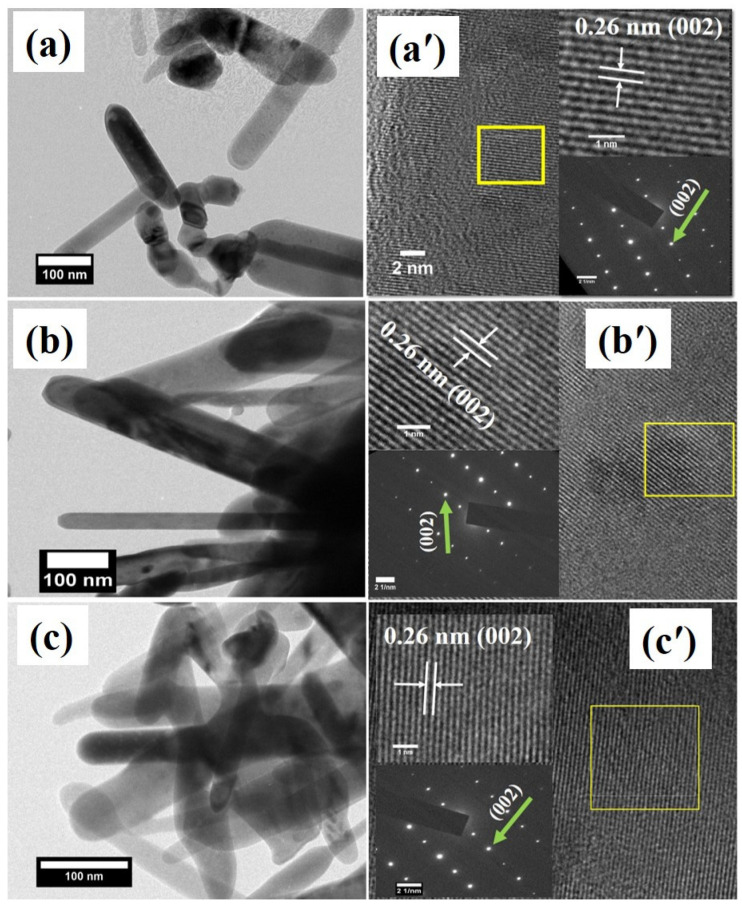
(**a**–**c**) TEM micrographs of Cu-doped ZnO nanostructures, (**a**′–**c**′) HR-TEM micrographs of Cu-doped ZnO nanostructures and insets represents the SAED pattern and zoom part of HR-TEM image.

Figure 4a–c demonstrates the morphology of the Cu-doped ZnO nanostructures that were studied by using TEM. The inset in Figure 4a′–c′ represents the HR-TEM micrograph and the SAED pattern of the Cu-doped ZnO nanostructures. The TEM images obviously infer that the Cu-doped ZnO nanostructures exhibit a rod-like nanostructure and are analogous to the FE-SEM results. The interplanar spacing which was calculated using HRTEM highlighted in Figure 4a′–c′ was found to be 0.26 nm for the un-doped and Cu-doped-ZnO nanostructures, and which is matched with the ZnO (002) plane. The SAED patterns were recorded by focusing the electron beam on an individual nanorod Cu-doped ZnO. The SAED pattern undoubtedly infers that the Cu-doped ZnO nanostructures display hexagonal crystal structures. The HR-TEM and SAED results are analogous to those of the XRD, and they infer the single crystalline nature of the Cu-doped ZnO nanostructures and exclude the presence of any secondary phase. 

UV-VIS spectroscopy was used to study the effect of Cu doping on the ZnO nanostructures. The bandgap energies (E_g_) of the Cu-doped ZnO nanostructures have been determined using Tauc’s equation: αhν=A(hν−Eg)12, where A is a constant, hν is the photon energy, h is Planck’s constant, and E_g_ is the optical band-gap energy. The E_g_ measured by Tauc’s plots is displayed in Figure 2d. The E_g_ value of the un-doped ZnO nanostructures was calculated from the intercept of the ${\left(\alpha h\nu \right)}^{2}$ versus (hν) curves, and it is found to be 3.38 eV. Here, it is worth to mention that the E_g_ value is measured as 3.33 eV and 3.23 eV for 1% and 5% the Cu-doped ZnO nanostructures, respectively. The decrease in the Eg with the Cu doping is due to the sp–d spin-exchange interactions between the band electrons as well as the localized d electrons of the Cu ion substituting the host cation. The bandgap narrowing is generally due to the s–d and p–d exchange interactions, which may cause a positive and a negative correction to the conduction- and the valence-band edges [17].

**Figure 5:**
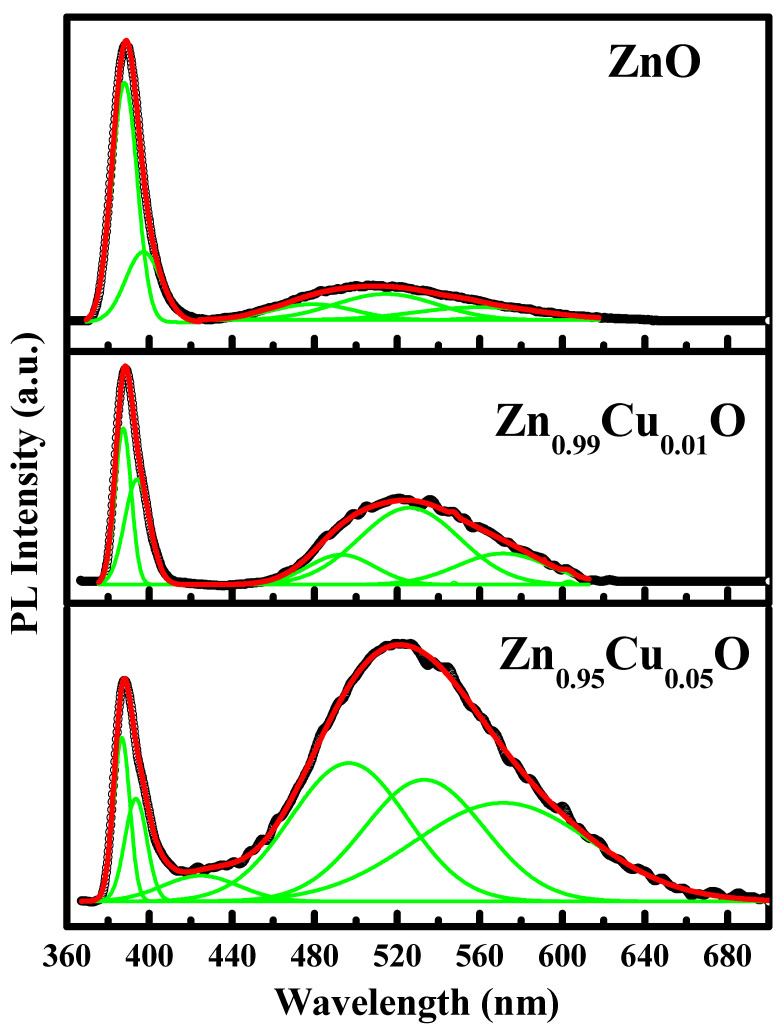
Room temperature photoluminescence spectra of Cu-doped ZnO nanostructures.

Figure 5 demonstrates the photoluminescence (PL) of the Cu-doped ZnO nanostructures that were measured at room temperature with an excitation wavelength of 325 nm. It is well known that the PL spectroscopy is an excellent technique that is used to study intrinsic and extrinsic defects, and it is a very important tool for understanding the structural imperfections in semiconductors. It can be seen from Figure 5 that the Cu-doped ZnO nanostructures show one high-intensity peak in the UV region, whereas the other peaks are in the blue-green region. To investigate the various emissions, the visible spectrum was analyzed using a Gaussian fitting. Two emission peaks at 387 and 397 nm can be observed in the NBE emission of the un-doped ZnO, and these peaks shifted towards the higher wavelength side after the Cu doping at 386 nm, and this was 394 nm for 1% Cu-doped samples, and 386 nm and 393 nm for 5% Cu-doped samples, thereby revealing the transitions from the surface trap to the VB and from Zn_i_ to the VB. It is evident from the literature that the high-intensity peak in the UV region originates due to the near-band-edge emission (NBE) transition in the band gap of ZnO. This NBE transition occurred because of the recombination of free excitons through an exciton–exciton collision process. It is worth mentioning that the oxygen vacancy or Zn interstitials are responsible for the blue-green emission, which is also known as deep-level emission (DLE). The DLE is accredited to the single ionized oxygen vacancy which results from the recombination of a photo-generated hole with the single ionized charged state of the defects in the Cu-doped ZnO nanostructures. Both the un-doped and Cu-doped samples exhibit blue/violet emission bands, and after the Cu doping in the ZnO lattice, a blue emission band can be observed to have emerged at 492 and 496 nm. The defects at the zinc interstitial site (Zn_i_) and the oxygen vacancy (V_o_) are the contributory factors to the PL emissions in Cu-doped ZnO. The zinc interstitial (Zn_i_) energy level and the zinc vacancy recombination generate the blue emissions at 492 nm and 496 nm (V_Zn_), repsectively. The blue emission peaks at 492 and 496 nm originate from an electron transition from the top of the valence band to the zinc interstitial (Zn_i_) site. The green emissions have two peaks at 514 and 560 nm in the spectral range of 514 to 571 nm. The green luminescence band at 514 nm is generated by the recombination of the electrons in singly ionized oxygen vacancies with photo-excited holes in the VB. After the Cu doping in the ZnO nanoparticles, the green emission peak shifts towards the higher wavelength side at 524, 533, 571, and 571 nm [3]. It is reported that the DLE in the Cu-doped ZnO is because of charge transfer between the Cu^2+^ ions and the neighboring oxygen atoms. It is observed that the intensity of the NBE peak decreases with the Cu doping, whereas that of blue-green emission increases. The increase in the intensity of the blue-green emission peaks clearly implies that the density of the defects increases with the Cu doping, and they are the highest for the 5% Cu-doped ZnO nanostructures. The PL spectra establish that the DLE shifts towards the higher wavelength with the Cu doping. In the past years, PL spectroscopy studies have been carried out by a number of research groups and they have reported different theories of the green emission in ZnO [27,28,29,30,31]. Leiter et al. have reported that a triplet excited state of neutral oxygen vacancy is responsible for the unstructured green emission in ZnO [32], but according to Reynold et al. [27], such a type of transition has resulted from the transition of two shallow donors to a deep acceptor. According Wang et al. [29], the positions and intensities of the blue-green emission change with the annealing time as well as the temperature. The redshift that was observed in the Cu-doped ZnO nanostructures inferred that the Cu ions were substituted for the tetrahedrally coordinated Zn ions in the ZnO host matrix. As the doping % of Cu increased, the intensity of the DLE emission also emerged, indicating that doping has a substantial influence on the formation of various defects in the ZnO lattice. 

**Figure 6:**
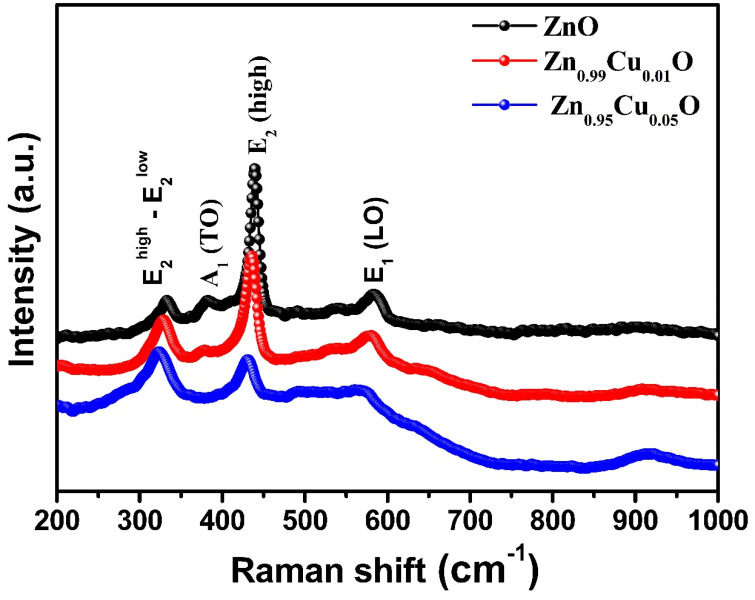
Raman spectra of un-doped and Cu-doped ZnO nanostructures.

Figure 6 shows the Raman spectra of the un-doped and Cu-doped ZnO nanostructures ranging from 200–1000 cm^−1^ to examine how the Cu dopant affects the Raman scattering in the doped samples. The highest intensity mode indicated as $E_{2}^{\mathrm{High}}$ at around 438 cm^−1^ corresponds to the characteristic band of ZnO with a hexagonal wurtzite geometry, and this might be attributed to the non-polar phonon vibrations of ZnO. As shown in Figure 6, the intensity of $E_{2}^{\mathrm{High}}$ decreases with the Cu doping concentration, indicating that the Cu-doped atoms degrade the crystal quality of ZnO. The peaks at around 332, 380, and 584 cm^−1^ are Raman modes that may be related to defect-induced modes [33]. The fact that the Cu addition enhances the tensile stress in the ZnO crystal and causes the E_2H_ mode to move towards a lower wave number signifies the occurrence of imperfections and voids. The transverse optical (TO) and longitudinal optical (LO) first-order vibrational bands, indicated as A_1_(TO) and E_1_(LO), respectively, are apparent on both sides of the characteristic band. The Raman spectra of the un-doped and Cu-doped ZnO exhibit a second-order (E_2H_–E_2L_) Raman mode and an A_1_(TO) symmetry mode at 332 cm^−1^ and 380 cm^−1^, respectively. As the Cu concentration in the samples increases, the intensity of the peak at 330 cm^−1^ increases, suggesting that the nanoparticles are single crystals. The E_1_(LO) mode, a short peak that can be seen at 584 cm^−1^ in ZnO and the Cu-doped nanoparticles, is supposed to be induced by defects such as oxygen vacancies. The E_1_(LO) mode broadens and shifts to a lower wave number with an increasing Cu concentration in the ZnO nanolattice, indicating an increase in the oxygen vacancies in the Cu-doped ZnO nanoparticles [34]. 

**Figure 7:**
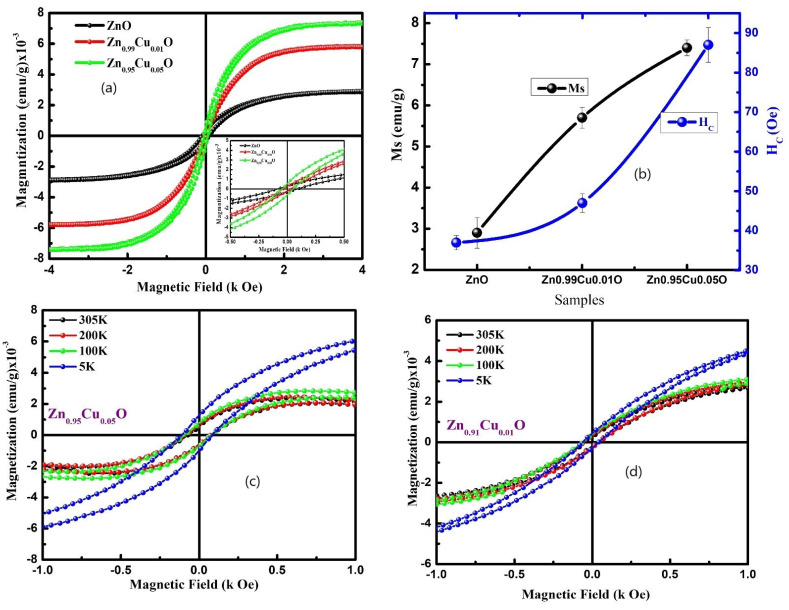
(**a**) M–H loop of Cu-doped ZnO nanostructures. (**b**) Coercive field (H_C_) and saturation magnetization (M_S_) of Cu-doped ZnO nanostructures. (**c**) Low temperature M–H loop Zn_0.99_Cu_0.01_O nanostructures. (**d**) Low-temperature M–H loop Zn_0.95_Cu_0.05_O nanostructures.

**Figure 8:**
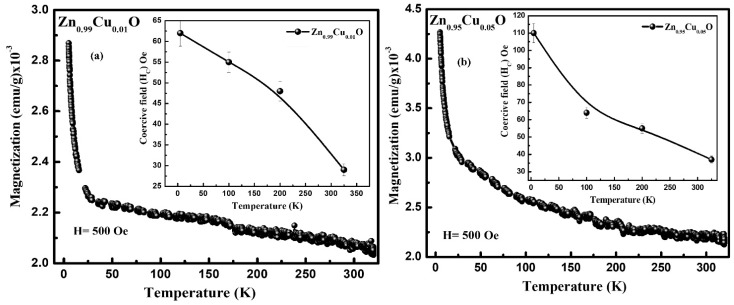
(**a**) Field cooled magnetization vs. temperature curve of Zn_0.99_Cu_0.01_O nanostructures. Inset shows the H_C_ of Zn_0.99_Cu_0.01_O nanostructures at different temperatures. (**b**) Field cooled magnetization vs. temperature curve of Zn_0.95_Cu_0.05_O nanostructures. Inset shows the H_C_ of Zn_0.95_Cu_0.05_O nanostructures at different temperatures.

The magnetic behavior of the Cu-doped ZnO nanostructures has been studied using the dc magnetization measurements. Figure 7a shows the magnetization (M) versus the magnetic field (H) curve of the Cu-doped ZnO nanostructures which were measured at room temperature. It can be seen from Figure 7a (see inset) that the un-doped and Cu-doped ZnO nanostructures show a ferromagnetic behavior at room temperature. The various magnetic parameters such as the remanence magnetization (M_R_), the coercive field (H_C_), and the saturation magnetization (M_S_) were evaluated from the M–H curves. The value of M_R_ for the un-doped ZnO nanostructures is 2.6 × 10^−4^ emu/g and 3.2 × 10^−4^ as well as 5.5 × 10^−4^ emu/g for the 1% and 5% Cu-doped ZnO nanostructures, respectively, showing an increasing trend with Cu doping. The values of M_S_ and H_C_ (see Figure 7b) are also found to increase from 2.9 × 10^−3^ to 7.4 × 10^−3^ emu/g and 37.0 Oe to 87.0 Oe, respectively, with the Cu doping in the ZnO nanostructures. Furthermore, we have performed the low-temperature magnetic measurement of the 1% and 5% Cu-doped ZnO nanostructures. The M–H curves were recorded at 5 K, 100 K, 200 K, and 305 K, and they have been displayed in Figure 7c,d. The low-temperature M–H curves demonstrate that the value of H_C_ decreases (see inset in Figure 8a,b) with an increase in the temperature. Additionally, the magnetization versus the temperature (M–T) measurements were carried out for the 1% and 5% Cu-doped ZnO nanostructures in the temperature range from 5 K to 305 K. The M–T curves were measured in the field-cooled (FC) mode. In the FC mode, the samples were cooled down from 300 K to 5 K in the presence of a magnetic field of 500 Oe, and their magnetization was recorded. It was noticed that magnetization started to decrease rapidly at the low temperatures until 25 K, and then, it decreases slowly. Many groups have reported the ferromagnetic behavior of the un-doped and TM-doped ZnO [18,19,21,22,23,35,36,37,38,39,40,41], regardless of its origin, is still unclear. Some groups have observed ferromagnetic ordering in the Cu-doped ZnO, and this has been explained using the carrier-mediated model [22], but others have discussed using the bound magnetic polarons (BMP) model [42]. Therefore, based on the literature, the ferromagnetic ordering in the Cu-doped ZnO may be explained by the BMP model. The Cu-doped ZnO nanostructures have the potential to be used in room-temperature-operated spintronics devices due to their weak ferromagnetism. In order to support the magnetic ordering at room temperature, the increase in the total magnetization with Cu doping demonstrates the significance of either the formation of oxygen vacancies or dopant-generated defects. A hysteresis loop and an s-shaped M–H curve at room temperature indicate the presence of a weak ferromagnetic order with paramagnetic activity. 

## 4. Conclusions

In summary, the Cu-doped ZnO nanostructures (Cu% = 0, 1, 5) which were prepared using a microwave-assisted chemical route synthesis are characterized by the x-ray diffraction (XRD), the field emission scanning electron microscopy (FE-SEM) and the energy dispersive x-ray (EDX) spectroscopy, the photoluminescence (PL) and the dc magnetization data in order to investigate the structural, morphological, optical and magnetic properties of them. The XRD, HR-TEM and SAED results validated that all of the samples exhibited a single-phase polycrystalline wurtzite hexagonal structure that is similar to the ZnO sample. The lattice parameters were observed to decrease as the Cu content increased. The morphological analysis revealed the formation of nanostructures showing manifestation of an increasing aspect ratio with an increasing Cu% content. The photoluminescence (PL) spectra indicate the photoemission shift with an increasing Cu% content in both the UV and blue-green regions. The PL spectra confirm that intrinsic defects persist in the samples, implying that structural modifications induce the lattice defects to evolve. Raman spectroscopy study demonstrated that the peak intensity of the $E_{2}^{\mathrm{High}}$ mode decreases and shifted to a lower wave number, confirming the degradation of the crystalline nature of the doped samples. The peak intensity of E_1_(LO) increases with the Cu doping, which suggests that the defects and oxygen vacancies have developed due to increased Cu doping in the ZnO nanostructure. Therefore, we can conclude from our PL and Raman results that increased Cu doping promotes additional defects and oxygen vacancies to form in the ZnO nanostructures. All of the samples exhibit increasing saturation magnetization and coercivity, thereby demonstrating ferromagnetic behavior. On the contrary, the temperature-dependent response reveals decreasing saturation magnetization and coercivity with increasing temperatures from 5 K to 305 K. In summary, the introduction of Cu ions in the ZnO lattice leads to the generation of intrinsic defects, which results in improved magnetic properties. These nanostructures were modified by Cu doping, which promises better yield samples with ferromagnetic properties for their potential uses in technological applications such as spintronics, optoelectronics, and photocatalysts.

## Figures and Tables

**Table 1:** Various structural parameters obtained from the Rietveld refinement and bandgap energy of Cu-doped ZnO nanostructures.

Sample	Lattice Parameters (Å)	Unit Cell Volume (Å^3^)	χ^2^	Rp	Rwp	Rexp	Bandgap (E_g_)
ZnO	a = b = 3.2506 c = 5.2070	47.650	1.87	5.89	8.15	7.82	3.38 eV
Zn_0.99_Cu_0.01_O	a = b = 3.2505 c = 5.2066	47.642	1.83	7.03	9.2	9.5	3.30 eV
Zn_0.95_Cu_0.05_O	a = b = 3.2500 c = 5.2058	47.621	1.22	6.27	5.99	8.9	3.23 eV

## Data Availability

Available on request.
